# Evaluating the impact of home supportive counseling and telephone supportive counseling on postpartum depression and anxiety: a randomized controlled trial

**DOI:** 10.1186/s12888-025-06953-7

**Published:** 2025-05-20

**Authors:** Bita Eskandari, Roghaiyeh Nourizadeh, Esmat Mehrabi, Rasoul Heshmati, Reyhaneh Ivanbagha, Zahra Akbarivand

**Affiliations:** 1 Student Research Committee, Department of Midwifery, Faculty of Nursing and Midwifery, Tabriz University of Medical Sciences, Tabriz, Iran; 2 Department of Midwifery, Faculty of Nursing and Midwifery, Tabriz University of Medical Sciences, Tabriz, Iran; 3 Women’s Reproductive Health Research Center, Al-Zahra Hospital, Tabriz University of Medical Sciences, Tabriz, Iran; 4 Faculty of Education and Psychology, University of Tabriz, Tabriz, Iran

**Keywords:** Supportive counseling, Postpartum depression, Postpartum anxiety

## Abstract

**Background:**

Childbirth and postpartum experience often lead to the significant physiological and emotional changes in mothers. Hormonal fluctuations, coupled with the adaptation to maternal roles, play a pivotal role in postpartum depression and anxiety. The present study aimed to evaluate the effect of home and telephone supportive counseling on postpartum depression and anxiety.

**Methods:**

This randomized controlled trial was conducted on 93 primiparous women aged 18–45 years attended the Taleghani and Al-Zahra educational and medical centers in Tabriz, Iran. Subjects were randomly assigned to the home supportive counseling, telephone supportive counseling, and control (receiving routine postpartum care) groups. Home and telephone supportive counseling were conducted for three 30–45 min sessions scheduled on postpartum days 3–5, 7–9, and 20–25. Data were collected using the Edinburgh Postnatal Depression Scale and the Postpartum Specific Anxiety Scale 10–15 days and 42–60 days postpartum, and analyzed using ANCOVA and Kruskal-Wallis tests.

**Results:**

The mean (SD) depression score was 6.23 (3.09) in the telephone supportive counseling group, 4.78 (3.85) in the home supportive counseling group, and 5.79 (3.39) in the control group during 10–15 days postpartum (*P* = 0.016). The mean (SD) anxiety score was 28.11 (9.37) in the telephone supportive counseling group, 27.32 (7.38) in the home supportive counseling group, and 39.88 (7.73) in the control group during 10–15 days postpartum (*P* < 0.001). However, no statistically significant difference in depression and anxiety scores was observed among the three groups during 42–60 days postpartum (*P* > 0.05).

**Conclusion:**

Home supportive counseling effectively alleviates symptoms of postpartum depression within 10–15 days postpartum. Moreover, both home and telephone supportive counseling were found to reduce postpartum anxiety symptoms within 10–15 days postpartum. Considering the cost-effectiveness of phone counseling, it is recommended that healthcare providers use telephone supportive counseling to reduce early postpartum anxiety.

**Registration clinical trials:**

Iranian Registery of Clinical Trials-Beta vertion, https://irct.behdasht.gov.ir/trial/71775 (IRCT20170506033834N11), registered 2023.8.20.

## Introduction

The postpartum period represents a critical phase in the lives of mothers and their newborns [1]. The World Health Organization defines this period as the interval from immediately after delivery to six weeks (42 days) postpartum [2]. Childbirth and the postpartum experience often lead to the significant physiological and emotional changes for mothers, especially primiparous mothers without prior experience, as adjusting to the maternal role and accepting the responsibility of newborn care can be challenging [3]. These difficulties may contribute to mental health issues such as stress, anxiety, and depression in postpartum women [4–6].

Approximately 10% of women experience depression during the first year postpartum, and nearly 20–40% of mothers suffer from mood disorders after childbirth [7, 8]. Postpartum depression is the most common psychological disorder during the postpartum, occurring at any time within the first year after birth. The risk of postpartum depression in the near miss mothers are even twice as likely as women with normal childbirth experience [9]. Studies indicated that adequate support can reduce the risk of postpartum anxiety and depression while enhancing mothers’ self-esteem [10, 11]. Maternal depression and anxiety increase the risk of adverse outcomes in infant feeding such as delayed initiation of breastfeeding and lack of breastfeeding continuity [12].

In Iran, mothers are typically discharged from the hospital shortly after childbirth, and spend the initial days and weeks of motherhood at home without the close support of healthcare providers [13]. In this regard, appropriate psychological support and supportive counseling, especially for primiparous mothers, seem essential. Supportive counseling aids individuals in organizing distressing emotional states, strengthening, and developing coping strategies. It emphasizes exploring the experiences, reactions, and emotions of clients, with a primary focus on self-esteem, ego functionality, and adaptability skills [14]. This counseling approach does not rely on a cohesive theory; rather, it is a set of techniques applied within any theoretical framework. This counseling approach is primarily focused on clarification and emotional catharsis [15].

A systematic review [16] assessed the effect of various home interventions on maternal and neonatal outcomes and found that the quality of evidence was low, and further well-designed trials were recommended to develop an optimal care package. Home support interventions can shift care towards a client-centered approach. Because home counseling increases costs, telephone consultation is considered as an effective means for providing consultation and facilitating access to care. The importance of telephone health counseling was highlighted during the COVID-19 pandemic. Lavender et al. conducted a review study on 12,256 women and reported ambiguous and inconclusive results regarding the effect of telephone support on postpartum anxiety, as data were collected at different time intervals using various assessment tools [17]. Considering the critical role of postpartum support, and the lack of comparative studies on telephone versus home counseling during postpartum, this study aimed to compare the effect of the telephone and home supportive counseling on postpartum depression and anxiety.

## Method

### Study design and participants

This randomized controlled trial (RCT) was conducted on 93 primiparous women delivered vaginally at Taleghani and Al-Zahra Educational and Medical Centers in Tabriz, Iran. The inclusion criteria consisted of primiparous women aged 18–45 years, singleton pregnancy, and mothers of full-term neonates weighing 2500–4000 g. The exclusion criteria encompassed mothers with hospitalized neonates, systemic or chronic diseases, or conditions exacerbated by pregnancy and childbirth such as gestational or overt diabetes, hypertension, or cardiac disorders. Additional exclusions included adverse life events within the past three months such as bereavement or divorce, a postpartum depression score of 13 or higher on the Edinburgh Postnatal Depression Scale, pre-existing or pregnancy-related depressive disorders, and neonatal abnormalities or mortality.

The sample size was calculated 28 per group using G-POWER software based on the findings of Bahari et al. [18] for the postpartum depression variable and considering M1 = 13.1, assuming a 20% reduction following the intervention, M2 = 10.48, SD1 = SD2 = 3.42, Power = 80%, and two-sided α = 0.05. Additionally, based on the study of Shamsdanesh et al. [19] for the anxiety variable and regarding M1 = 48.65, assuming 20% post-intervention reduction, M2 = 38.92, SD1 = SD2 = 12.75, Power = 80%, and two-sided α = 0.05, the sample size was estimated to be 28 per group. Accounting for a 10% attrition, the final sample size was adjusted to 31 subjects per group.

### Sampling

This study was commenced following ethical approval from the Ethics Committee of Tabriz University of Medical Sciences and registration in the Iranian Registry of Clinical Trials (IRCT). A total of 93 primiparous women, aged 18 to 45 years, delivered vaginally previous day, were recruited from Taleghani and Al-Zahra Educational and Medical Centers in Tabriz, Iran. The researcher attended the centers, identified eligible mothers, and provide information about the study objectives.

The day after delivery, written informed consent form was obtained from women at the hospital, followed by completing the demographic characteristics profile and the Edinburgh Postnatal Depression Scale. They were then randomly assigned to the home supportive counseling, telephone supportive counseling, and control (receiving routine postpartum care) groups with a ratio of 1:1:1 using block randomization with block size of 3 and 6. For allocation concealment, assignments were written on paper and placed in sequentially numbered opaque envelopes, which were opened in order by non-involved individual in sampling process. In this study, neither the subjects nor the researcher were blinded due to the nature of the study, and only the data collector was blinded to group allocation.

### Data collection tools

The data were collected using the demographic characteristics profile, the Edinburgh Postnatal Depression Scale, and the Postpartum Specific Anxiety Scale.

### Demographic characteristics profile

This form encompassed questions regarding subjects’ age, educational level, occupation, income, infant gender, and additional relevant factors.

### Edinburg postnatal depression scale (EPDS)

The Edinburgh Postnatal Depression Scale, developed by Cox et al. [20], is employed to measure depression during pregnancy and postpartum. The scale consists of ten four-option questions, ranked in ascending severity for some items (1, 2, and 4) and descending severity for others (3, 5, 6, 7, 8, 9, and 10). Each response is scored on a scale of 0–3, reflecting the severity of symptoms, and the total score, ranging from 0 to 30, is calculated by summing the scores of all items. The validity of this scale was evaluated 0.78 through concurrent correlation coefficient of EPDS and the Beck Depression Inventory. The reliability of the EPDS was estimated to be 0.75 using Cronbach’s alpha and the split-half method. Montazeri et al. [21] reported the Cronbach’s alpha for the Iranian version of the scale, ranged between 0.77 and 0.86, with a correlation coefficient of 0.80.

### Postpartum specific anxiety scale research short-form (PSAS-RSF)

The Postpartum Specific Anxiety Scale was initially developed and psychometrically validated by Davies et al. [22]. The scale consisted of 51 items and measured the subdomains of maternal competence and attachment anxiety, infant safety and welfare concerns, practical infant care anxiety, and psychosocial adjustment to motherhood. Due to the large number of items, a shorter version was later developed and psychometrically validated and this study utilized the 16-item version. The PSAS-RSF is the first validated short form for assessing postpartum-specific anxiety, which can be used up to 12 months postpartum. Each subdomain includes four items, with response options ranging from “never” to “almost always,” scored from one to four, respectively and the total score range from 16 to 64 [23]. Hassanzadeh et al. [24] reported a Cronbach’s alpha of 0.93 and an intraclass correlation coefficient (ICC) of 0.92 for the Iranian version of the scale.

### Intervention

An introductory session was held for subjects at the hospital on the day following delivery. A home supportive counseling was conducted for three 30–45 min sessions scheduled on postpartum days 3–5, 7–9, and 20–25. In the second intervention group, telephone support counseling was provided through the same schedule and session duration. The timing of each session was coordinated with the subjects in advance.

Supportive counseling strategies include establishing a counselor-client alliance, accepting the client’s perceptions, assisting the client in expressing emotions, clarifying ambiguities, linking the client’s circumstances to their emotions and behaviors, enhancing and promoting social support, reinforcing positive thoughts, and helping the client discover solutions. The counseling center is to reduce stress by providing information about the challenges the patients face in adapting to their situation [25]. In this approach, deep exploration of conflicts and profound interpretations are not conducted, and the emphasis is on the clarification and emotional release [26].

The counseling sessions were structured in accordance with the support counseling protocol [25, 26] and focused on encouraging subjects to share their experiences, express their concerns and difficulties encountered in self-care and infant care, expressing and reflecting on the mother’s emotional and affective responses to the new circumstances, exploring functional disturbances, signs such as stress and incorrect adaptive skills by the counselor to identify the mothers’ weaknesses and adaptive skills, teaching mothers coping strategies based on the problem solving such as positive thinking, teaching relaxation exercises, supporting clients in exploring solutions such as seeking and securing social support, and enhancing interpersonal communication skills, especially with their partners. All counseling sessions were conducted by the first author under the supervision of the fourth author.

The control group received only routine postpartum care. Anxiety and depression scales (10–15 days and 42–60 days postpartum) were completed by all three groups in Taleghani and Al-Zahra obstetric clinics. The sixth author collected the data and individuals with serious problems were identified and referred to a psychiatrist.

### Data analysis

The collected data were analyzed using SPSS version 25. The normality of data distribution was assessed using the Shapiro-Wilk test. Further, Kruskal-Wallis test were utilized to compare maternal depression scores among the three groups during 10–15, and 42–60 days postpartum and ANCOVA was employed for comparing the anxiety scores during 10–15 and 42–60 days postpartum. All analyses were performed based on the intention-to-treat (ITT) principle.

## Results

**Fig. 1:**
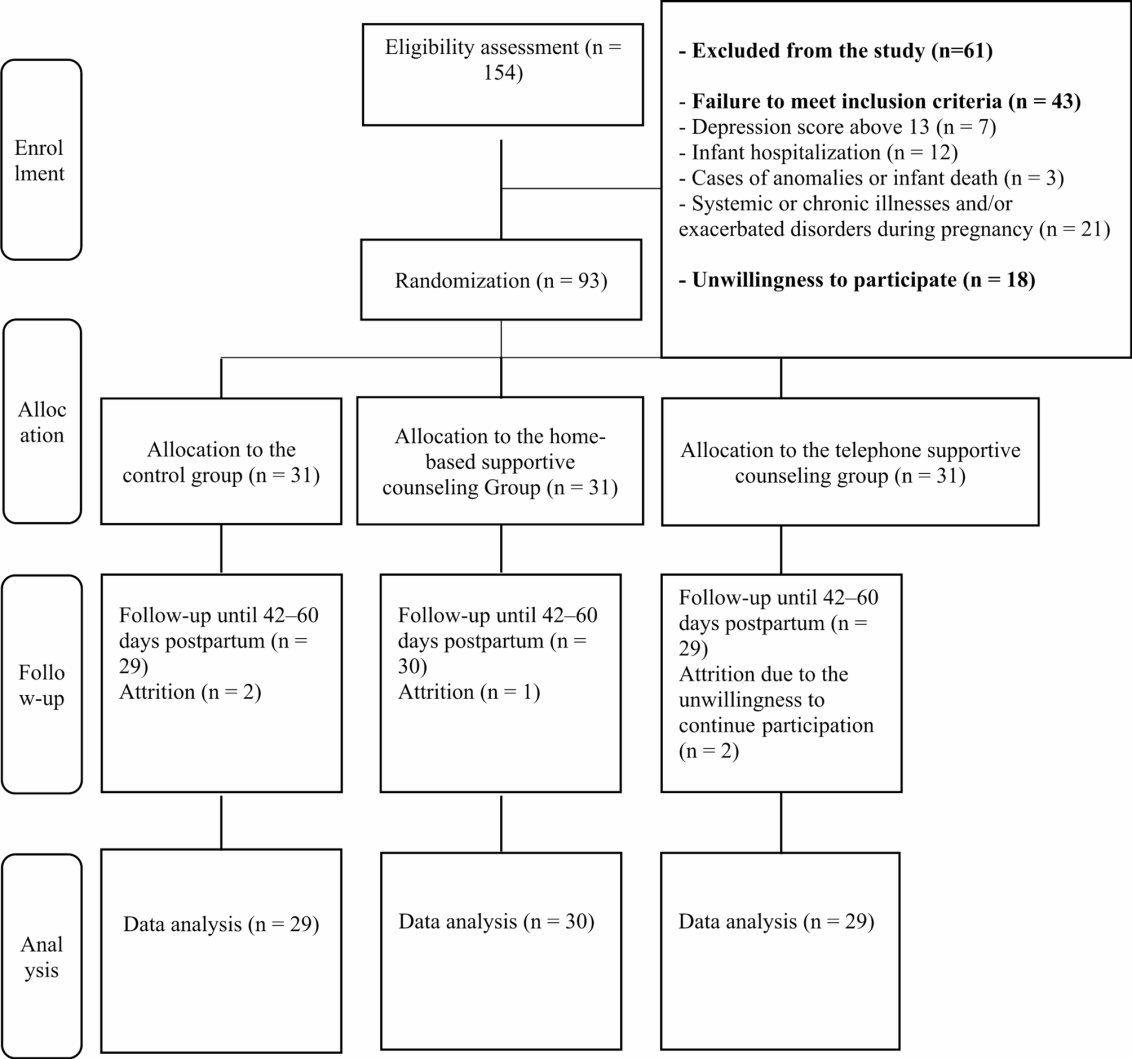
Flowchart of the study

This study was conducted on primiparous women in Tabriz, Iran, from October 6, 2023, to August 21, 2024. A total of 154 postpartum women were screened, of whom 61 women were excluded either for being ineligible or unwilling to participate. Ultimately, 93 women who had vaginal delivery were enrolled in the study and were randomly allocated to one of three groups of telephone supportive counseling, home supportive counseling, and control. During the study, two subjects from the telephone supportive counseling group, one from the home supportive counseling group, and two from the control group were excluded due to the unwillingness to continue (Fig. 1). There was no statistically significant difference in the demographic characteristics among these groups, except for their educational level (Table 1).

**Table 1:** The demographic characteristics of the participants (*n* = 93)

Variable	Telephone supportive counseling (*n* = 31) No (%)	Home supportive counseling (*n* = 31) No (%)	Control group (*n* = 31) No (%)	*p*
**Age (Years) †**	23.14 (5.91)	22.97 (4.88)	23.00 (4.97)	0.991*
**Educational level**				0.045**
Elementary/Guidance school University	20 (64.5)	17 (54.8)	10 (33.3)	
High School/Diploma	9 (29.0)	11 (35.5)	16 (50.0)	
University	2 (6.4)	3 (9.7)	5 (16.7)	
**Occupation**				1.00**
Housekeeper	30 (96.8)	30 (96.8)	(100.0) 31	
Employed	(3.2) 1	(3.2) 1	0 (0.0)	
**Housing Status**				0.506**
Personal	(20.0) 6	(27.6) 8	(33.3) 10	
Rental	(73.3) 23	(62.1) 20	(53.3) 17	
Living with relatives	(6.7) 2	(10.3) 3	(13.3) 4	
**Income sufficiency**				0.440**
Sufficient	(19.4) 6	(13.3) 4	(24.1) 8	
Somewhat sufficient	(71.0) 22	(66.7) 20	(51.7) 15	
Insufficient	(9.7) 3	(20.0) 6	(24.1) 8	
History of infertility	(3.3) 1	(3.3) 1	(9.6) 3	0.60**
**Fetal gender**				0.194**
Female	(48.4) 15	(45.2) 14	(66.7) 21	
Male	(51.60 16	(54.8) 17	(33.3) 10	

†Mean (SD), *One-way ANOVA, **Chi-square test

Before the intervention and after adjusting the effect of educational level, ANCOVA test revealed no statistically significant difference among three groups (*P* = 0.496). However, a statistically significant difference was observed during 10–15 days postpartum (*P* = 0.016); as the mean depression score in the home supportive counseling group was lower than that in the other two groups. During 42–60 days postpartum, no statistically significant difference in the mean depression score was found among three groups (*P* = 0.341) (Table 2).

**Table 2:** Comparing the mean depression score among three groups during 10–15 days and 42–60 days postpartum

Groups	Home supportive counseling (*n* = 30) Mean (SD)	Telephone supportive counseling (*n* = 29) Mean (SD)	Control (*n* = 29) Mean (SD)	*P*
**Before intervention†** **(Score range: 0–30)**	8.36 (3.99)	8.07 (3.66)	6.56 (2.69)	0.496*
**10–15 days postpartum†** **(Score range: 0–30)**	4.78 (3.85)	6.23 (3.09)	5.79 (3.39)	0.016**
**42–60 days postpartum†** **(Score range: 0–30)**	2.48 (3.08)	2.18 (2.91)	3.95 (2.38)	0.341**

*Analysis of covariance after controlling for the confounding factor, **Kruskal-Wallis test

The mean (SD) anxiety score was 28.11 (9.37) in the telephone supportive counseling group, 27.32 (7.38) in the home supportive counseling group, and 39.88 (7.73) in the control group during 10–15 days postpartum (*P* < 0.001); as both intervention groups demonstrated lower mean anxiety score compared to the control group. During 42–60 days postpartum, no statistically significant difference was seen in terms of anxiety among the groups (*P* = 0.379) (Table 3).

**Table 3:** Comparing the mean anxiety score among three groups during 10–15 days and 42–60 days postpartum

Groups	10 to 15 days postpartum (Score range: 16–64) Mean (SD)		42 to 60 days postpartum (Score range: 16–64) Mean (SD)	
Telephone supportive counseling (*n* = 29)	28.11 (9.37)		18.04 (1.77)	
Home supportive Counseling (*n* = 30)	27.32 (7.38)		18.67 (3.20)	
Control (*n* = 29)	39.88 (7.73)		17.93 (1.78)	
P*	0.001>		0.379	

Comparison of Groups	Mean Difference (95% Confidence Interval)	P*	Mean Difference (95% Confidence Interval)	P*
Phone supportive counseling vs. control	-11.77 (-16.78 to -6.77)	0.001>	0.10 (-1.33 to 1.55)	0.997
Home supportive counseling vs. control	-12.55 (-17.42 to -7.69)	0.001>	0.73 (-0.61 to 2.08)	0.462
Phone supportive counseling vs. home support counseling	0.783 (-3.75 to 5.31)	0.966	-0/62 (-2.11 to 0.86)	0.668

*Analysis of Covariance (ANCOVA) after controlling for the effect of confounding variables

## Discussion

This is the first study worldwide examined the impact of home supportive counseling and telephone supportive counseling on postpartum depression and anxiety. The results revealed that home supportive counseling significantly reduced postpartum depression compared to the telephone support counseling and control groups during 10–15 days postpartum. However, no statistically significant difference in postpartum depression scores was observed among the three groups during 42–60 days postpartum.

The results of this study are consistent with the results of prior research demonstrating the ineffectiveness of interventions for postpartum depression during 2–3 months postpartum [27–29].

Inconsistent with the results of present study, Milani et al. [30] conducted a RCT on 276 postpartum women in Tehran, Iran and found a significant improvement in depression level of the intervention group compared to the control group after receiving home care for two months. This discrepancy can be attributed to the difference in the intervention type (home-care package) applied in the study of Milani et al. compared to the home support counseling employed in the present study.

Further, Vismara et al. [31] examined the impact of the reflective parenting home visiting program on 77 primiparous mothers with healthy infants in Italy and revealed a significant decrease in depression levels among mothers in the intervention group compared to those in control group during 3- to 12-month postpartum, which is not in line with the results of the present study. The difference in the results may be due to the difference in the content and approach of the intervention used in Vismara et al.‘s study.

In another study, Dennis et al. [32] evaluated the effectiveness of peer telephone support to prevent postnatal depression on 701 women at high risk for depression in Canada and reported a 64% reduction in the relative risk of depression during 12-week postpartum, which is not consistent with the results of present study. This discrepancy can be attributed to population and the nature of the intervention (peer support), which was delivered by a community-recruited volunteer who had previously experienced and recovered from postpartum depression and had participated in a 4-hour training session.

Contrary to the results of the present study, Chyzzy and Cindy [33] assessed the impact of peer telephone support on postpartum depression among pregnant adolescents aged 16–24 in Toronto. The results demonstrated that adolescents in the intervention group, who received peer support from a trained peer mentor through mobile communication (calls or text messages) during their third trimester and 12 weeks postpartum, had significantly lower depression scores at 12-week postpartum compared to the control group. The difference may be explained by variations in the target population and the type of intervention (peer support).

The results of this study indicated that both home supportive counseling and telephone supportive counseling led to a reduction in postpartum anxiety compared to the control group during 10–15 days postpartum. However, no statistically significant difference in postpartum anxiety score was observed among these groups during 42–60 days postpartum.

Consistent with the findings of this study, Simhi et al. [34] demonstrated that the WAWA (What Am I Worried About? ) intervention, accompanied by a self-help workbook addressing common anxieties experienced by new mothers and incorporating cognitive-behavioral and mindfulness techniques, significantly reduced postpartum anxiety and stress scores after the intervention. The subjects opted the telephone support reported a more substantial reduction in anxiety and stress scores compared to those selected group intervention.

In addition, Koçak et al. [35] in their study sought to design a mobile support application to assist postpartum mothers and evaluate its effects on maternal anxiety level. They concluded that although the postpartum mobile support application provided a valuable and reliable source of information, it was not effective in reducing anxiety during the six-week postpartum evaluation, which is in line with the findings of present study.

In a systematic review, Lavender et al. [17] evaluated the impact of telephone support during pregnancy and the first six-week postpartum on maternal and neonatal outcomes. The review included data from 27 RCTs involving 12,256 women. There was no strong evidence to confirm a reduction in maternal anxiety during pregnancy or postpartum following telephone support. The interpretation of the results regarding postpartum anxiety was unclear, as the data were collected at different time points using various instruments.

Since there was no significant difference between the home and telephone supportive counseling groups in anxiety mean score and considering the cost-effectiveness of phone counseling to in-home one, it is recommended that health care providers utilize telephone supportive counseling to reduce early postpartum anxiety.

### Strengths and limitations

One of the notable strengths of this study was the random allocation of participants to groups and allocation concealment to eliminate the selection bias, whereas limitations included the lack of blinding of participants due to the nature of the study and reliance on self-reported data. Moreover, the study was conducted in a single geographical region. The small sample size was another limitation of the present study. In addition, this study was conducted on primigravida women, and the results cannot be generalized to multiparous women.

## Conclusion

Supportive counseling provided at home appears to be effective in alleviating symptoms of postpartum depression within the first two weeks after delivery. Evidence indicates that both in-home and telephone-based counseling approaches can help decrease anxiety levels during this early postpartum period. Due to its affordability and practicality, telephone counseling emerges as a viable strategy for addressing early postpartum anxiety. In addition, further efforts should focus on implementing mental health support programs with a long-term perspective, and future research should explore these interventions specifically in women who have undergone cesarean delivery to enable comparison of results.

## Data Availability

The data sets used and/or analyzed during the present study are available from the corresponding author on reasonable request.
